# Melatonin Enhances Thermal Resilience and Extends Worker Lifespan in *Apis cerana* via Redox–Metabolic Reprogramming

**DOI:** 10.3390/insects17040379

**Published:** 2026-04-01

**Authors:** Ke Wang, Lianjun Zhou, Xianfu Xiang, Miaomiao Wei, Chenglian Lu, Wenfeng Li, Richou Han, Yi Zhang

**Affiliations:** 1 Key Laboratory of State Administration of Traditional Chinese Medicine for Production & Development of Cantonese Medicinal Materials, Guangzhou Comprehensive Experimental Station of National Industrial Technology System for Chinese Materia Medica, Guangdong Engineering Research Center of Good Agricultural Practice & Comprehensive Development for Cantonese Medicinal Materials, School of Chinese Materia Medica, Guangdong Pharmaceutical University, Guangzhou 510006, China; 18868014696@163.com (K.W.); zlj200117@163.com (L.Z.); xxf18699050228@163.com (X.X.); weimiaomiao2002@163.com (M.W.); lcl202505@163.com (C.L.); 2 Guangdong Key Laboratory of Animal Conservation and Resource Utilization, Guangdong Public Laboratory of Wild Animal Conservation and Utilization, Institute of Zoology, Guangdong Academy of Sciences, Guangzhou 510260, China; wfli.bees@giz.gd.cn (W.L.); hanrc@giz.gd.cn (R.H.)

**Keywords:** *Apis cerana*, over-summering, melatonin, redox homeostasis, detoxification enzymes

## Abstract

*Apis cerana* is widely managed in apiculture in Southern China but experiences substantial colony losses during prolonged summer heat. Developing effective strategies to support colony over-summering is therefore critical. This study demonstrates that dietary supplementation with melatonin significantly enhances thermal tolerance and extends worker lifespan in *A. cerana* under heat stress. Laboratory bioassays revealed that melatonin supplementation (20 µg/mL) markedly improved worker survival at both 35 °C and 37 °C, with the most pronounced effect at 37 °C, where mortality was significantly reduced. Consistently, field trials demonstrated that melatonin supplemented colonies gained significantly more weight during summer heatwaves than colonies without melatonin supplementation. Mechanistically, melatonin orchestrates a biphasic adaptive response. In an early phase (day 4), melatonin rapidly upregulates heat shock proteins (*HSC70-4*, *CRYAA*, *l(2)efl*) and detoxification enzymes (*GST-like*), accompanied by reduced reactive oxygen species (ROS) accumulation and enhanced proboscis extension response (PER), indicative of preserved sensory function. This is followed by a later maintenance phase (day 11), characterized by sustained upregulation of fatty acyl-CoA reductases (*FAR1*, *FAR11-like*, *FARwat*) and peroxisomal components (*PMP34*), which promote lipid remodeling and membrane stabilization. RNA-seq analysis identified differentially expressed genes (DEGs) significantly enriched in pathways related to redox homeostasis, lipid metabolism, detoxification (*GSTs*, *CarEs*, *CYP450s*), and longevity. These molecular responses were associated with enhanced antioxidant capacity, reduced oxidative damage, and sustained foraging activity under thermal stress. Collectively, these results indicate that melatonin serves as a potent nutritional intervention that reprograms redox metabolic networks to mitigate heat-induced damage, extend worker longevity, and enhance colony productivity under climate warming. These findings highlight melatonin’s potential as a practical tool to reduce summer colony losses in apiculture.

## Simple Summary

Honey bees, particularly the indigenous *Apis cerana*, face escalating threats from prolonged summer heat waves in southern China, a challenge intensified by climate change. Current apicultural practices lack effective strategies to mitigate heat-induced colony losses. In this study, we demonstrate that dietary melatonin supplementation significantly improves heat tolerance and extends worker lifespan under both laboratory-controlled (35 °C and 37 °C) and field summer heat conditions. Melatonin-treated colonies exhibited greater weight gain during heat waves, indicating enhanced colony productivity. Using an integrated approach combining survival assays, behavioral tests, biochemical analyses, and time-resolved transcriptomics, we reveal an early protective phase that reduces oxidative damage and helps preserve sensory function, followed by a later maintenance phase that supports lipid remodeling and membrane stability. These findings position melatonin as a practical and low-cost nutritional intervention to bolster honey bee resilience against thermal stress, an increasingly critical need in the context of global warming.

## 1. Introduction

*Apis cerana*, the indigenous honey bee of China, has been domesticated for over 2000 years [1]. In the early 20th century, the introduction of *Apis mellifera* together with modern beekeeping technologies spurred significant advancements in apiculture, leading to increased production of honey, royal jelly, pollen, and other hive products [2]. Despite these developments, *A. cerana*—owing to its unique biological traits—remains dominant in traditional apiculture, particularly in remote mountainous regions [3]. Compared with *A. mellifera*, *A. cerana* exhibits several adaptive advantages, including heightened olfactory sensitivity, efficient utilization of sparse floral resources, robust foraging capacity, lower food consumption, and greater resilience to both biotic and abiotic environmental stresses [3,4,5]. In recent years, the Chinese government has actively promoted the conservation and management of *A. cerana* as a regionally important pollinator species in Southern China. China spans approximately 5500 km from north to south, encompassing a latitudinal range of over 50°. This extensive geographic range gives rise to pronounced climatic heterogeneity. In southern China, beekeeping is challenged by prolonged heat exposure, with elevated temperatures persisting for up to eight months per year—peaking between July and September [4]. During this period, ambient temperatures frequently exceed 35 °C for more than two consecutive weeks, and the frequency and intensity of extreme heat events are increasing under climate change [6,7]. These conditions pose significant challenges to *A. cerana*. Studies show that queen oviposition declines markedly at 35 °C and is nearly suppressed above 38 °C [8,9]. In response, worker bees engage in wing fanning, hive ventilation, and water collection to thermoregulate the colony, maintaining an internal temperature of approximately 35 °C [8,10,11]. However, these thermoregulatory mechanisms have inherent physiological and behavioral limits [8,9]. Prolonged exposure to temperatures exceeding the colony’s buffering capacity can trigger absconding or emergency swarming. Moreover, sustained thermoregulation is energetically demanding, resulting in reduced worker longevity, impaired immunity, and diminished capacity for disease resistance and predator defense, ultimately contributing to colony collapse [12]. Developing effective strategies to mitigate heat stress, therefore, remains a critical challenge for the apiculture sector.

Heat stress adversely affects key biological processes in honey bees, including development, behavior, and immunocompetence [13]. It induces excessive accumulation of reactive oxygen species (ROS), causing oxidative damage to macromolecules, including DNA, proteins, and lipids, and disrupting cellular homeostasis, as documented in insects such as *Drosophila melanogaster* [8,12,14,15,16]. In response, antioxidant defense systems are activated, including the upregulation of key enzymes such as catalase (CAT), which scavenge ROS and mitigate oxidative damage, thereby helping to maintain cellular homeostasis.

In addition to antioxidant enzymes, detoxification enzymes including glutathione S-transferases (GSTs), carboxylesterases (CarEs), cytochrome P450 monooxygenases (CYP450s), and acetylcholinesterase (AChE) contribute to resilience under heat stress by neutralizing cytotoxic metabolites [15,17,18,19]. To cope with heat stress, insects reallocate energy toward heat shock protein (HSP) synthesis at the expense of somatic maintenance and growth [20]. This resource trade-off depletes lipid and protein reserves while elevating carbohydrate stores such as hemolymphatic trehalose [10,21]. Prolonged resource depletion, however, accelerates aging and shortens lifespan. For instance, *A. mellifera* under heat stress exhibits metabolic dysregulation that compromises long-term survival [1,9,22]. Notably, supplementation with extrogenous antioxidant can reduce ROS accumulation and extend lifespan by restoring redox homeostasis [23]. Melatonin (N-acetyl-5-methoxytryptamine) was first isolated from the bovine pineal gland and named for its ability to induce skin lightening in tadpoles by dispersing melanophores [24]. It is an evolutionarily conserved molecule that functions as a hormone in animals and a signaling compound in plants and microbes, whose biosynthesis is tightly regulated by photoperiod [25]. Melatonin critically modulates circadian rhythms, immune responses, and antioxidant defenses, thereby maintaining physiological homeostasis and enhancing resilience to environmental stressors [25,26,27]. It is well known for its anti-aging properties, which arise from its ability to directly scavenge reactive oxygen and nitrogen species (ROS/RNS), including H_2_O_2_, •NO, ONOO^−^, O_2_•^−^, ROO•, and •OH, in both lipophilic and hydrophilic cellular compartments [23,28].Owing to its pleiotropic biological functions, exogenous melatonin has been widely applied across biomedical, agricultural, and entomological contexts [29,30,31]. In biomedicine, exogenous melatonin is used to bolster immune function and counteract the effects of aging in humans and animals. In livestock production, it modulates nutrient partitioning, growth, and seasonal reproductive cycles [22,31,32]. In crop production, exogenous melatonin enhances plant resilience to biotic and abiotic stresses [33]. Exogenous melatonin has also been explored in entomology [14]. Dietary melatonin extends developmental duration in *D. melanogaster* [14]. In *A. cerana*, it enhances cold tolerance and modulates behavior following ingestion [34].

Here, we evaluate melatonin as a dietary intervention to enhance antioxidant capacity and survival of *A. cerana* under heat stress. We quantify dose and temperature-dependent effects on worker survival and integrate these phenotypic outcomes with host transcriptome profiling to elucidate the underlying mechanisms. At both individual and colony levels, we further assessed gustatory responsiveness (via the proboscis extension response, PER) and colony weight gain during summer heatwaves to evaluate the behavioral and energetic correlates of melatonin-mediated protection.

## 2. Materials and Methods

### 2.1. Honey Bees

*A. cerana* colonies were maintained on the campus of Guangdong Pharmaceutical University in Yunfu, Guangdong Province, China. This subspecies is neither endangered nor protected under Chinese law. To minimize genetic and environmental variability, all experimental bees were sourced from a single apiary. Newly emerged workers from this apiary were used for laboratory cage assays, while independent colonies from the same apiary were enrolled in the field trial.

### 2.2. Laboratory Bioassays of Melatonin Treatment Under Controlled Temperatures

Frames containing sealed brood were removed from healthy colonies and placed individually in mesh-walled cages and maintained in an insect growth chamber at 34 ± 1 °C and 55 ± 5% relative humidity (RH) [2]. After incubation overnight, newly emerged workers were collected, pooled, and randomly assigned to experimental treatments.

To evaluate whether melatonin mitigates heat stress and whether its effects are dose-dependent, bees were exposed to three temperature regimes: 33 °C, 35 °C, and 37 °C (all at 55% RH). Within each temperature group, bees were fed 50% (*w*/*v*) sucrose syrup mixed with melatonin (Purity 98%, Macklin, Cas No. 73314) at concentrations of 0 (negative control), 4, 12, or 20 µg/mL. Each treatment group comprised six replicate cup cages (35 bees per cage). The cage was made of plastic with 8 cm diameter and 14cm high, and a top hole was made for syrup feeding, two side holes for ventilation. Three cages were dedicated for survival monitoring, and three were used for sample collection for molecular analyses. Syrup (3 mL) was provided via 10 mL syringe feeders and replaced every three days. Cages were housed in separate insect incubators set to their respective experimental temperatures. Dead bees were removed and counted daily until one treatment group reached 100% mortality.

For transcriptomic analysis, bees were sampled on day 4 (D4) and day 11 (D11) post-treatment. At each time point, five bees were randomly selected from each of the three biological replicates per treatment (*n* = 15 bees per group). Of these, five bees were randomly allocated for RNA sequencing (RNA-seq), and the remaining nine were used for RT-qPCR validation.

### 2.3. Proboscis Extension Response (PER) and Oxidoreductase Activity Assays

To evaluate changes in gustatory responsiveness and physiological robustness following melatonin ingestion, a PER assay together with enzymatic activity measurements was performed [35]. Newly emerged workers were collected as above, then pooled and divided into four treatment groups, each group were treated separately as below, Group I, 35 °C + 20 µg/mL melatonin, Group II, 35 °C + 0 µg/mL melatonin (control), Group III, 37 °C + 20 µg/mL melatonin, and Group IV, 37 °C + 0 µg/mL melatonin (control). After four days of treatment, the healthy and active worker bees were selected for PER testing, reactive oxygen species (ROS) quantification, and oxidoreductase activity assays [36]. For the PER assay, worker bees were starved for 18 h and then individually restrained in modified 1.5 mL microcentrifuge tubes. Fifteen minutes prior to testing, antennal responsiveness was confirmed by stimulation of 50% sucrose; individuals that failed to respond were excluded [35]. During the assay, both antennae were sequentially stimulated with sucrose solutions of increasing concentration (0.1%, 0.3%, 1%, 3%, 10%, and 30%) using a 2.5 µL pipette tip. A full proboscis extension was scored as a positive response. Each treatment consisted of three biological replicates, 20 bees per replicate, resulting in a total of 60 bees per treatment. Hemolymph was collected from the thorax of 50 bees per group using disposable 20 µL glass capillaries and kept on ice. Heads were dissected on a chilled plate, pooled by group (50 bees), and homogenized in ice-cold buffer. Both hemolymph and tissue homogenates were centrifuged at 12,000 rpm for 5 min at 4 °C. The resulting supernatants were diluted 1:4 (*v*/*v*) with phosphate-buffered saline (PBS), and total protein concentration was determined using a BCA-based Total Protein Assay Kit (Nanjing Jiancheng Bioengineering Institute, Cat. No. A045-4-2, Nanjing, China).

Activities of glutathione S-transferase (GST), carboxylesterase (CarE), and cytochrome P450 (CYP450) were measured using commercial ELISA kits (Nanjing Jiancheng Bioengineering Institute) following the manufacturer’s protocols. Absorbance was read at 450 nm using a microplate reader, and enzyme activities were expressed as units per milligram of protein. ROS levels were quantified using a ROS assay kit (Nanjing Jiancheng, Cat. No. E004-1-1) with fluorescence intensity measured at 488 nm and normalized to µg of protein.

### 2.4. Field Colony Trial

A one-month field intervention was conducted from July to August 2024. Eighteen *A. cerana* colonies of comparable strength were randomly assigned to two groups (*n* = 9 per group). Healthy colonies were selected based on the absence of obvious disease symptoms and normal colony performance before the experiment. Colonies in the treatment group received pollen patties supplemented with melatonin at 200 mg per 100 g pollen (*w*/*w*), which were freshly prepared and replaced weekly. Colonies in the negative control received identical pollen patties without melatonin. Consumption of the pollen patties was assessed by weighing the patties at each replacement, and no obvious refusal of the melatonin-supplemented patties was observed during the experimental period. All colonies were additionally provided with 50% sucrose syrup every three days. Hive weights were recorded on July 1 and August 1, and colony weight gain (kg/colony) was used as a proxy for metabolic performance during summer heat stress.

### 2.5. High-Throughput RNA Sequencing and Bioinformatic Analysis

Total RNA was extracted from adult *A. cerana* workers and used to construct strand-specific, paired-end RNA-seq libraries, which were sequenced on an Illumina platform. Raw FASTQ files were processed with fastp to remove adapters, trim low-quality bases, and generate Q20/Q30 quality metrics. Clean reads were aligned to the *A. cerana* reference genome (NCBI RefSeq assembly GCF_029169275.1, AcerK_1.0 with corresponding GTF annotation) using STAR under default parameters. Alignment quality including mapping rate, genomic distribution, splice junction accuracy, strand specificity, and gene-body coverage—was evaluated with RSeQC.

Transcript assemblies were generated per sample using StringTie (v2.1.7), merged across samples, and classified with gffcompare (v0.12.6). Novel multi-exon transcripts ≥200 bp were retained for downstream analyses. Coding sequences were predicted with TransDecoder (v5.7.1), and the longest open reading frame per transcript was annotated via BLASTx against the SWISS-PROT database (E-value ≤ 1.0 × 10^−3^). Functional annotations were assigned based on Gene Ontology (GO), Pfam, and SUPERFAMILY databases. Gene-level expression was quantified using StringTie (v2.1.7) and reported as both fragments per kilobase of transcript per million mapped reads (FPKM) and raw read counts. Sample relationships were assessed via pairwise Pearson correlations and principal component analysis (PCA) based on log_2_ (FPKM + 1) values. Differentially expressed genes (DEGs) were identified using DESeq2 (v1.40.2), with significance defined as |log_2_ (fold change)| ≥ 1 and false discovery rate (FDR)-adjusted *p* < 0.05. Alternative splicing events were compared between groups, and functional enrichment of DEGs was performed using PANTHER and DAVID, with GO and KEGG pathways corrected for multiple testing via the Benjamini–Hochberg procedure.

### 2.6. RT-qPCR Validation

A subset of DEGs associated with the “longevity regulating pathway—multiple species” and “peroxisome” pathways was selected for RT-qPCR validation. Gene expression was assessed in bees sampled on D4 and D11 at 35 °C and 37 °C, comparing control (0 µg/mL) and melatonin-treated (20 µg/mL) groups. *β-actin* and *GAPDH* were used as reference genes. Primer sequences for target and reference genes are listed in Table 1. Amplification efficiencies for all target and reference genes were confirmed to be comparable (slope of normalized ΔCt vs. log input RNA ≤ 0.1 or efficiencies within ~90–110%). Reactions were performed using a SYBR Green qPCR Kit (TransGen Biotech, Cat. No. AQ601, Beijing, China) following the manufacturer’s instructions. Relative gene expression was calculated using the comparative Ct (ΔΔCt) method [37]. ΔCt was computed as ΔCt = average Ct_target_ − average Ct_β-actin._ The calibrator group (lowest expression level) was assigned a value of 1, and fold-change relative to the calibrator was calculated as 2^−ΔΔCt^. Data are presented as mean ± standard error (SE).

### 2.7. Statistical Analysis

All statistical analyses were performed using SPSS v27.0 and GraphPad Prism v9.0. Survival data were analyzed using Kaplan–Meier estimators and differences among groups were assessed using the log-rank (Mantel–Cox) test. When early survival differences were of particular interest, the Gehan–Breslow–Wilcoxon test was used and trends across ordered melatonin doses were examined using a log-rank test. Unless otherwise specified, tests were two-tailed with α = 0.05, and multiplicity-adjusted *p* values are reported where applicable. For comparisons involving more than three groups, one-way ANOVA was used to assess differences in gene expression among the four experimental groups, followed by Tukey’s HSD test for post hoc pairwise comparisons. Data are presented as mean ± standard deviation (SD), with n denoting the number of biological replicates.

## 3. Results

### 3.1. Melatonin Significantly Extended Honeybees Lifespan Under Heat Stress

**Figure 1:**
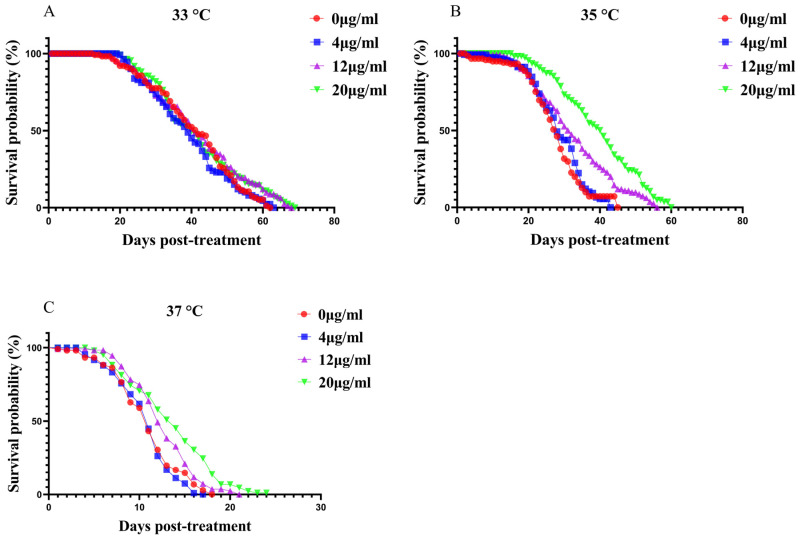
Temperature-dependent effects of melatonin on worker survival. Kaplan–Meier survival curves for *Apis cerana* workers kept at (**A**) 33 °C, (**B**) 35 °C and (**C**) 37 °C and fed sucrose syrup containing 0, 4, 12 or 20 µg mL^−1^ melatonin (*n* = 105 bees per dose at each temperature). Survival probability (%) is plotted against days post-treatment. Group differences were assessed by log-rank tests.

Mortality analysis was conducted to evaluate the effects of heat stress and the combined influence of heat stress alone and in combination with melatonin on the lifespan of *A. cerana* workers. As shown in Figure 1C, heat stress significantly reduced bee survivorship in a temperature-dependent manner. Bees exposed to 37 °C without melatonin supplementation exhibited the highest mortality, reaching 100% by day 18 post-treatment. In contrast, bees maintained at the optimal temperature of 33 °C (Figure 1A) showed a survival rate of 97.3% at the same time point. Under heat stress (37 °C), dietary melatonin markedly improved survival, with the most pronounced protective effect observed at the highest tested concentration (20 µg mL^−1^). This improvement was highly significant across all statistical tests: log-rank χ^2^ = 42.12, *p* < 0.0001; test for trend χ^2^ = 51.36, *p* < 0.0001; Gehan–Breslow–Wilcoxon χ^2^ = 32.99, *p* < 0.0001. Even under moderate heat stress (35 °C), melatonin conferred a substantial survival advantage (Figure 1B). Untreated bees reached 100% mortality by day 45, whereas bees receiving 20 µg mL^−1^ melatonin exhibited significantly extended lifespans (log-rank χ^2^ = 84.85, *p* < 0.0001; test for trend χ^2^ = 88.19, *p* < 0.0001; Gehan–Breslow–Wilcoxon χ^2^ = 66.13, *p* < 0.0001). Importantly, melatonin itself exerted no adverse effects on bee survival under non-stress conditions (33 °C). No significant differences in mortality were observed among groups receiving 0, 4, 12, or 20 µg mL^−1^ melatonin (log-rank χ^2^ = 3.023, *p* = 0.0821; test for trend χ^2^ = 6.132, *p* = 0.1053; Gehan–Breslow–Wilcoxon χ^2^ = 2.370, *p* = 0.4993), indicating that melatonin is non-toxic and well tolerated across the tested concentrations.

### 3.2. Melatonin Enhances Colony Weight Gain During Periods of High Temperature

**Figure 2:**
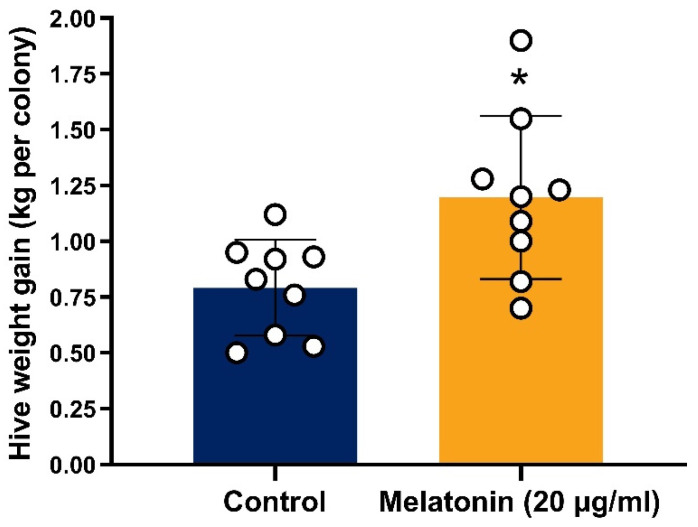
Colony weight gain during summer heat stress. * *p* < 0.05.

A field trial was conducted to evaluate the effect of melatonin supplementation under natural summer conditions. As shown in Figure 2, colonies receiving melatonin-supplemented pollen patties exhibited a significantly greater weight gain compared to control colonies during the high-temperature period (July–August) (*t*-test, *p* < 0.05; Table S1). 

Hive weights were measured before and after a one-month period in the hot season (July–August) to assess colony performance under field high-temperature conditions. Melatonin-treated colonies gained significantly more weight than controls (two-tailed *t*-test, *p* < 0.05). Bars show mean values and error bars indicate standard deviations (*n* = 9 hives per group) * *p* < 0.05.

### 3.3. Dietary Melatonin Preserves Sucrose Sensitivity Under Heat Stress

**Figure 3:**
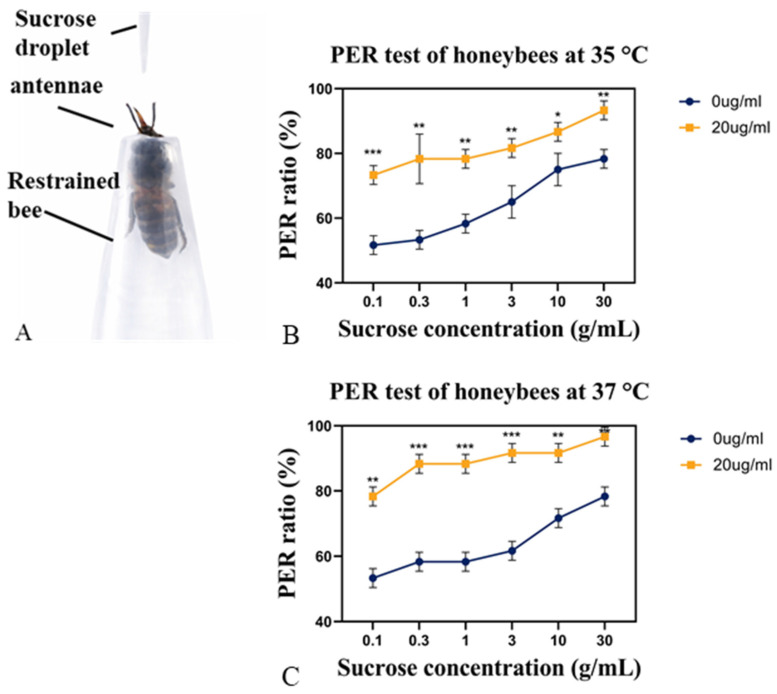
Melatonin enhances proboscis extension response (PER) under heat stress. (**A**) PER assay setup. (**B**,**C**) PER ratios of *Apis cerana* workers fed melatonin (20 μg/mL) or control sucrose solution and tested at 35 °C or 37 °C. Data = mean ± SD (*n* = 20). Statistical significance was determined using two-tailed *t*-tests (* *p* < 0.05, ** *p* < 0.01, *** *p* < 0.001).

To explore potential behavioral and physiological correlates of the increased colony weight gain, we assessed gustatory responsiveness using the proboscis extension response (PER) assay (Figure 3A; Table S2).

As shown in Figure 3B,C, melatonin-fed bees exhibited significantly higher PER responsiveness compared to control bees after five days of treatment at both 35 °C and 37 °C. At 35 °C, when stimulated with 0.1 g/mL sucrose, 73% of melatonin-treated bees responded with a PER, compared to only 51% the control group. PER responsiveness increased with sucrose concentration in both groups. However, melatonin-treated group consistently exhibited higher response rates. At the highest concentration tested (30 g/mL), 93% of melatonin-fed bees showed a PER, whereas only 78% of untreated group showed a response. The protective effect of melatonin on gustatory sensitivity was even more pronounced under severe heat stress (37 °C, Figure 3C). At sucrose concentration of 0.1 g/mL, PER response rates reached 78% in the melatonin-treated group, compared with 53% in controls. Similarly, at the sucrose concentration of 30 g/mL, 96% of melatonin-treated bees exhibited a PER, whereas only 78% of untreated bees responded. Across all sucrose concentrations, the difference in PER responsiveness between treated and control groups was greater at 37 °C than at 35 °C, indicating that melatonin helps preserve sensory sensitivity as thermal stress intensifies. These results suggest that dietary melatonin not only extends worker longevity but also maintains or enhances sucrose sensitivity during heat stress, which may support or aging behaviors and contribute to the observed increase in colony weight gain during summer conditions.

### 3.4. Dietary Melatonin Modulates Antioxidant and Detoxification Enzyme Activities Under Heat Stress

**Figure 4:**
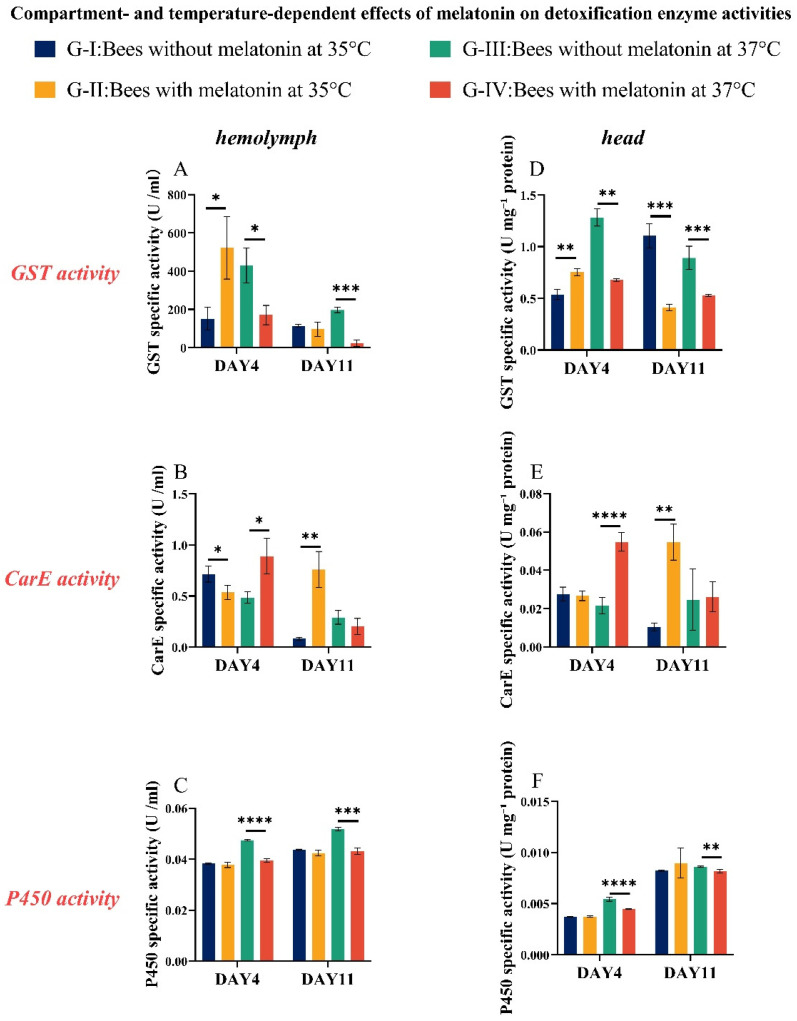
Effects of melatonin on detoxification enzyme activities under heat stress. Adult *Apis cerana* workers were maintained at 35 °C or 37 °C and fed sucrose syrup containing either 0 µg/mL (Negative Control) or 20 µg/mL melatonin. Enzyme activities were measured in hemolymph (**A**–**C**) and pooled head (**D**–**F**) on Day 4 (D4) and Day 11 (D11). Panels show specific activities of glutathione S-transferase (GST; **A**,**D**), carboxylesterase (CarE; **B**,**E**), and cytochrome P450 (CYP450; **C**,**F**). Hemolymph activities are expressed as U/mL; head–midgut activities are expressed as U/mg protein. Bars represent mean ± SD (*n* = 3 cages per treatment). Asterisks denote significant differences between melatonin-treated and corresponding control groups at the same temperature and sampling day (* *p* < 0.05, ** *p* < 0.01, *** *p* < 0.001, **** *p* < 0.0001; two-tailed unpaired *t*-tests).

The activities of three key detoxification and antioxidant enzymes—glutathione S-transferase (GST), carboxylesterase (CarE), and cytochrome P450 (CYP450)—were measured in both hemolymph and head tissues of *A. cerana* workers. As shown in Figure 4, the overall response patterns of these enzymes were consistent across the two tissue types under thermal stress (Table S3).

#### 3.4.1. Glutathione S-Transferase (GST)

At day 4 (D4), bees maintained at 35 °C and fed melatonin (20 µg mL^−1^) exhibited significantly elevated GST activity (521.82 U/mL^−1^) than control bees fed only sucrose syrup (151.72 U/mL; *t*-test, *p* = 0.0208), indicating that melatonin enhances GST activity under moderate heat stress. In contrast, at 37 °C, melatonin supplementation led to a significant reduction in GST activity (170.82 U/mL) relative to controls (430.29 U/mL; *t*-test, *p* = 0.0128). This suggests that while severe heat stress alone strongly induces GST, melatonin modulates this response, potentially preventing excessive or maladaptive activation. By D11, GST activity declined to below 200 U/mL in three of the four treatment groups; only bees exposed to 37 °C without melatonin maintained elevated levels, consistent with sustained oxidative pressure.

#### 3.4.2. Carboxylesterase (CarE)

CarE activity displayed a pattern distinct from GST. At D4 and 35 °C, melatonin-fed bees showed significantly lower CarE activity (0.54 U/mL) than controls (0.71 U/mL; *t*-test, *p* = 0.0415), suggesting melatonin may suppress CarE under moderate stress. In contrast, under severe heat stress (37 °C), melatonin supplementation significantly increased CarE activity to 0.89 U/mL, compared with 0.48 U/mL in controls (*t*-test, *p* = 0.0184). Together, these results indicate that melatonin differentially modulates CarE activity in a temperature-dependent manner—reduce activity at 35 °C while enhancing it at 37 °C—potentially reflecting context-dependent metabolic adjustment under heat stress.

#### 3.4.3. Cytochrome P450 (CYP450)

CYP450 activity remained relatively stable (~0.04 U/mL) in bees held at 35 °C across both D4 and D11, regardless of melatonin treatment. However, under 37 °C heat stress, CYP450 activity was significantly induced, reaching 0.055 U/mL in control bees—significantly higher than in melatonin-treated bees (0.045 U/mL; *t*-test, *p* < 0.05). This implies that extreme heat upregulates CYP450, and melatonin partially attenuates this induction, possibly by reducing oxidative or xenobiotic stress.

In short, these enzyme activity profiles demonstrate that melatonin exerts temperature-dependent, enzyme-specific modulation of the bee antioxidant and detoxification systems which enhances protective responses under moderate stress while preventing excessive activation under severe heat stress.

### 3.5. Effects of Melatonin on the ROS Scavenging and the Antioxidant Defense of Bees Exposed to High Temperature

**Figure 5:**
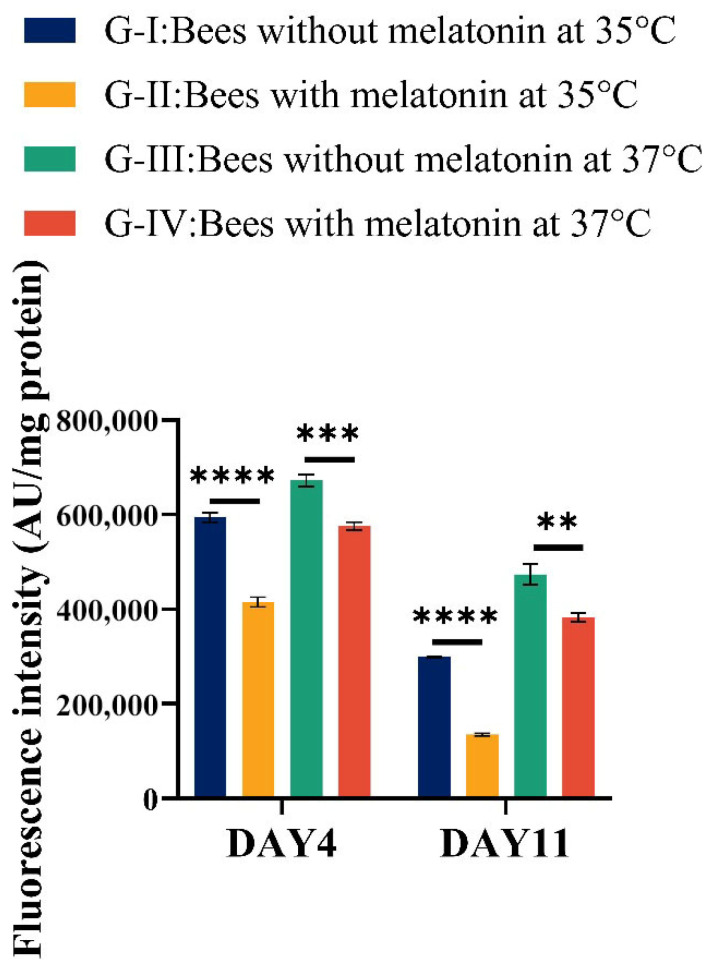
Melatonin suppresses heat-induced oxidative stress in *A. cerana* workers. Reactive oxygen species (ROS) levels, expressed as fluorescence intensity per milligram of protein (AU/mg protein), were measured in hemolymph and head of newly emerged workers maintained at 35 °C or 37 °C with or without dietary melatonin (20 µg/mL). Groups: G-I, 35 °C control; G-II, 35 °C + melatonin; G-III, 37 °C control; G-IV, 37 °C + melatonin. Data are shown as mean ± SEM (*n* = 50) Statistical significance: ** *p* < 0.01, *** *p* < 0.001 (two-tailed *t*-test). Melatonin significantly reduced ROS accumulation at both time points (D4 and D11), with pronounced effects under severe heat stress (37 °C), **** *p* < 0.0001.

To investigate the functional link between antioxidant enzyme activity and oxidative damage, reactive oxygen species (ROS) levels were quantified in experimental bees (Figure 5; Table S3). At day 4 (D4), ROS accumulation was significantly higher in bees exposed to severe heat stress (37 °C; Group III) than in bees maintained at 35 °C (Group I) (Group III vs. Group I, *p* < 0.001). Critically, melatonin supplementation markedly reduced ROS levels under both thermal conditions: Bees in Group II (35 °C + melatonin) exhibited significantly lower ROS than Group I (*p* < 0.001), and bees in Group IV (37 °C + melatonin) showed significantly lower ROS level than those in Group III (*p* < 0.001). These results demonstrate that melatonin effectively mitigates oxidative stress across both moderate and severe heat stress conditions. By D11, ROS levels had declined across all treatment groups, possibly reflecting physiological adaptation or metabolic recovery over time. Nevertheless, bees maintained at 37 °C (Groups III and IV) still displayed higher ROS levels than those at 35 °C (Groups I and II), confirming the persistent oxidative burden imposed by severe heat stress. Importantly, within the 37 °C cohort, melatonin-treated bees (Group IV) exhibited significantly lower ROS than their untreated counterparts (Group III, *p* < 0.01), indicating that melatonin provides sustained antioxidant protection even under prolonged and intense thermal challenge.

Together, these findings show that dietary melatonin significantly reduces oxidative damage in *A. cerana* under both moderate (35 °C) and severe (37 °C) heat stress. The protective effect of melatonin was more pronounced at high-temperature conditions and became increasingly evident over time, aligning with its role in modulating antioxidant enzyme activities and preserving cellular homeostasis.

### 3.6. Differential Expressed Genes (DEGs) Revealed Pathways and Genes Associated with Heat Stress and Melatonin

Qualified paired-end reads from cDNA libraries of four groups honeybees were obtained and were identified 1704 unique DEGs (|log_2_FC| > 1; FDR ≤ 0.05). To assess the effect of heat stress, gene expression profiles of bees incubated at different temperatures were compared. In bees not treated with melatonin, a total of 974 DEGs (327 upregulated and 647 downregulated) were identified at D4, and 1053 DEGs (648 upregulated and 405 downregulated) were detected at D11 when comparing bees without melatonin incubated at 37 °C with those maintained at 35 °C (Group III vs. Group I). Further analysis revealed that melatonin treatment significantly altered gene expression in a temperature- and time-dependent manner. At 35 °C, comparison of melatonin-treated and untreated bees identified170 DEGs (53 upregulated and 117 downregulated) at day 4 (D4), and 694 DEGs (344 upregulated and 353 downregulated) at day 11 (D11) (Group II vs. Group I). In contrast, at 37 °C, melatonin treatment resulted in 651 DEGs (199 upregulated and 452 downregulated) at day 4 (D4), and 186 DEGs (87 upregulated and 99 downregulated) at day 11 (D11) when compared with untreated bees (Group IV vs. Group III). The differentially expressed genes are summarized in the Supplementary Tables. It is noted that at the optimal temperature (35 °C), the number of DEGs increased with prolonged melatonin treatment, whereas under severe heat stress (37 °C), a larger number of DEGs was detected at the early time point (D4), followed by a marked reduction at D11.

**Figure 6:**
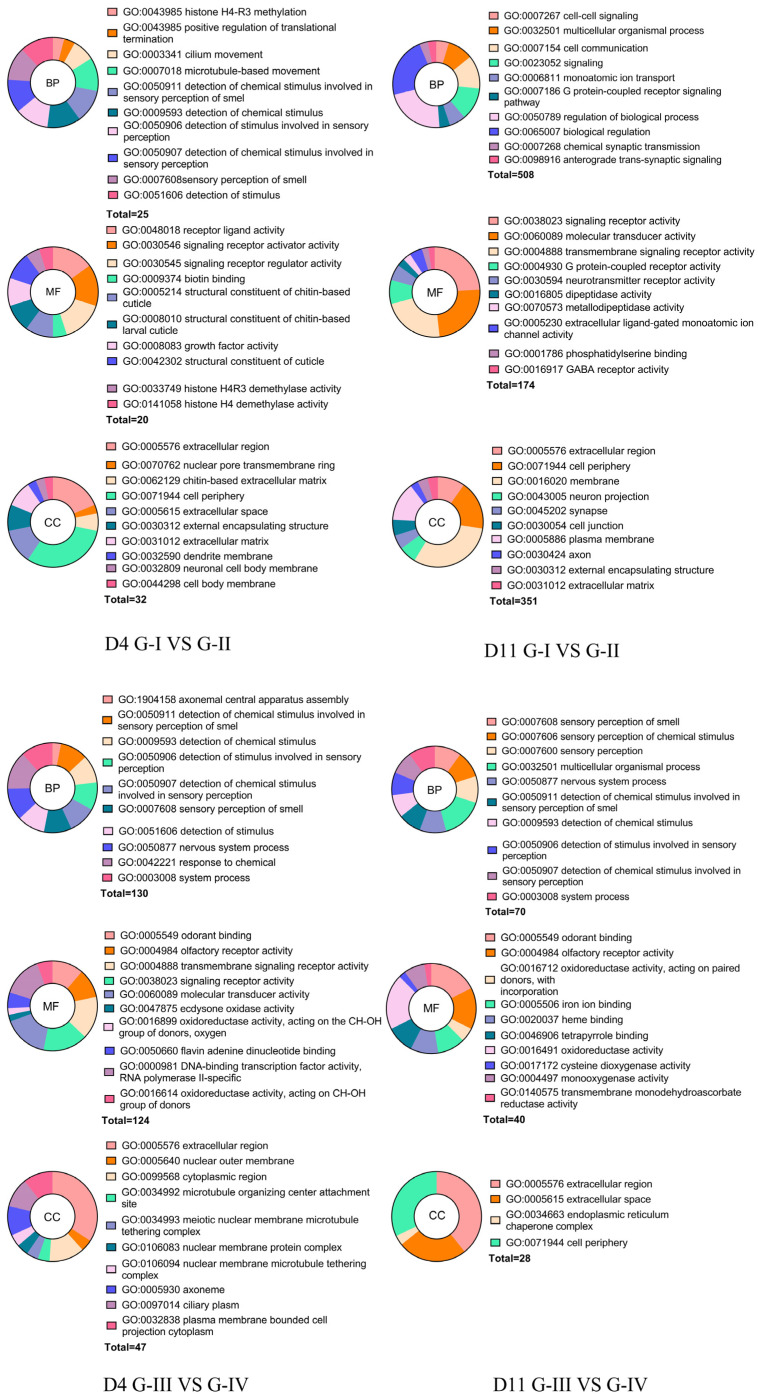

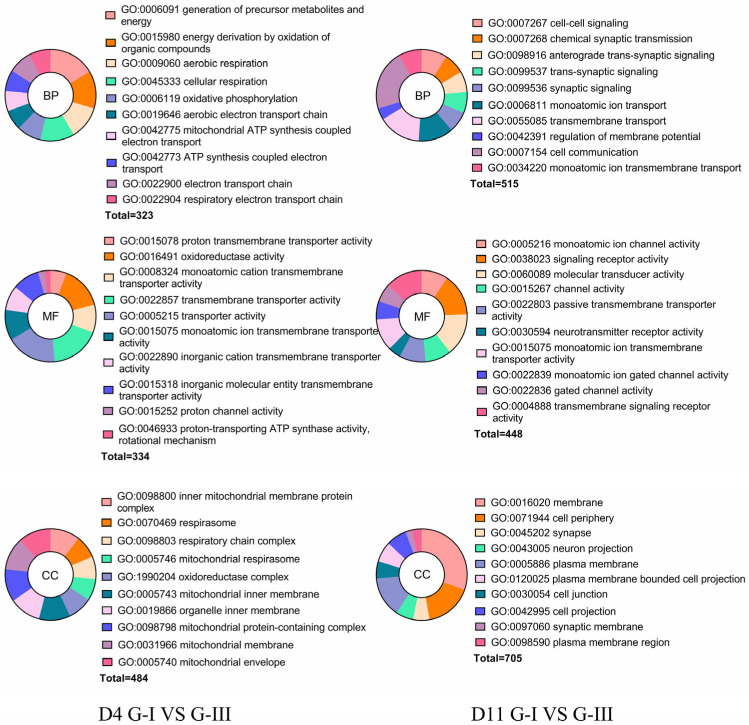
Gene Ontology (GO) enrichment of DEGs under temperature and melatonin treatments. Adult worker bees were assigned to four groups: G-I, Bees without melatonin at 35 °C; G-II, Bees with melatonin at 35 °C (20 µg/mL); G-III, Bees without melatonin at 37 °C; G-IV, Bees with melatonin at 37 °C (20 µg/mL). Samples were collected on Day 4 (D4) and Day 11 (D11). Differentially expressed genes (DEGs) were identified with DESeq2 (Benjamini–Hochberg–adjusted *p* ≤ 0.05 and |log_2_FC| ≥ 1). For each paired contrast (G-I vs. G-II and G-III vs. G-IV at D4/D11), DEGs were annotated to GO and enriched terms were summarized by ontology: biological process (BP), cellular component (CC) and molecular function (MF). The donut charts display the top enriched terms (FDR < 0.05); slice size reflects enrichment magnitude (e.g., GeneRatio/−log_10_FDR), and colors correspond to the listed terms.

To elucidate the functional implications of melatonin-induced transcriptional changes under heat stress, all differentially expressed genes (DEGs) from the six libraries were annotated against the Gene Ontology (GO) database and categorized into three ontologies: Biological Process (BP), Molecular Function (MF) and Cellular Component (CC) (Figure 6).

By day 4 (D4; Group III vs. Group I; 974 DEGs), the top three significantly enriched BP terms were generation of precursor metabolites and energy (GO: 0006091), energy derivation by oxidation of organic compounds (GO:0015980), and cellular respiration (GO: 0045333). The top three significantly enriched MF terms were transporter activity (GO: 0005215), transmembrane transporter activity (GO: 0022857) and oxidoreductase activity (GO: 0016491). The top three significantly enriched CC terms were inner mitochondrial membrane protein complex (GO: 0098800), respirasome (GO: 0070469), and respiratory chain complex (GO: 0098803). By D11 (Group-I vs. Group-III, 1053 DEGs), the top three significantly enriched BP terms were cell communication (GO: 0007154), transmembrane transport (GO: 0055085), and monoatomic ion transport (GO: 0006811). The top three significantly enriched MF terms were signaling receptor activity (GO: 0038023), transmembrane signaling receptor activity (GO: 0004888), and molecular transducer activity (GO: 0060089). The top three significantly enriched CC terms were membrane (GO: 0016020), cell periphery (GO: 0071944), and synapse (GO: 0045202).

At the early stage (D4, Group-I vs. Group-II, 170 DEGs), the top three significantly enriched BP terms were histone H4-R3 methylation (GO: 0043985), microtubule-based movement (GO: 0007018), and detection of chemical stimulus involved in sensory perception of smell (GO: 0050911). The top three significantly enriched MF terms were receptor ligand activity (GO: 0048018) and structural constituent of cuticle (GO: 0042302). The top three significantly enriched CC terms were extracellular region (GO: 0005576), nuclear pore transmembrane ring (GO: 0070762), and chitin-based extracellular matrix (GO: 0062129). By D11 (G-I vs. G-II, 697 DEGs), the top three significantly enriched BP terms were cell–cell signaling (GO: 0007267), biological regulation (GO: 0065007), and regulation of biological process (GO: 0050789). The top three significantly enriched MF terms were signaling receptor activity (GO: 0038023), molecular transducer activity (GO: 0060089), and transmembrane signaling receptor activity (GO: 0004888). The top three significantly enriched CC terms were extracellular region (GO: 0005576), cell periphery (GO: 0071944), and membrane (GO: 0016020).

In the parallel comparison (Group-III vs. Group-IV), early-stage enrichment at D4 (651 DEGs), the top three significantly enriched BP terms were response to chemical (GO: 0042221), system process (GO: 0003008), and nervous system process (GO: 0050877). The top three significantly enriched MF terms were molecular transducer activity (GO: 0060089), signaling receptor activity (GO: 0038023) and transmembrane signaling receptor activity (GO: 0004888). The top three significantly enriched CC terms were extracellular region (GO: 0005576), nuclear outer membrane (GO: 0005640), and cytoplasmic region (GO: 0099568). By D11 (G-III vs. G-IV, 186 DEGs), the top three significantly enriched BP terms were multicellular organismal process (GO: 0032501), sensory perception (GO: 0007600), and sensory perception of chemical stimulus (GO: 0007606). The top three significantly enriched MF terms were oxidoreductase activity (GO: 0016491), odorant binding (GO: 0005549) and olfactory receptor activity (GO: 0004984). The top three significantly enriched CC terms were extracellular region (GO: 0005576), extracellular space (GO: 0005615), and endoplasmic reticulum chaperone complex (GO: 0034663).

**Figure 7:**
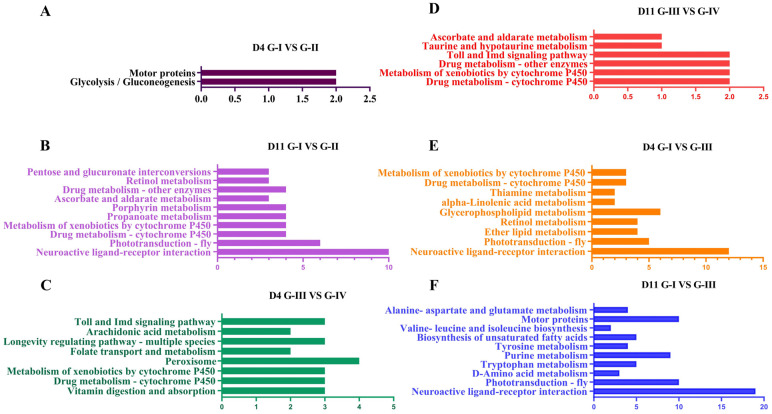
KEGG pathway enrichment of differentially expressed genes under melatonin and temperature treatments. Adult *Apis cerana* workers were assigned to four treatment groups: G-I, Bees without melatonin at 35 °C; G-II, Bees with melatonin at 35 °C (20 µg/mL); G-III, Bees without melatonin at 37 °C; G-IV, Bees with melatonin at 37 °C (20 µg/mL). Bees were sampled on Day 4 (D4) and Day 11 (D11), and differentially expressed genes (DEGs) were identified with DESeq2 (Benjamini–Hochberg FDR ≤ 0.05, |log_2_FC| ≥ 1). Bars depict the top enriched KEGG pathways for each pairwise comparison; bar length indicates the enrichment score (−log_10_FDR). (**A**) D4 G-I vs. G-II (melatonin effect at 35 °C); (**B**) D11 G-I vs. G-II; (**C**) D4 G-III vs. G-IV (melatonin effect at 37 °C); (**D**) D11 G-III vs. G-IV; (**E**) D4 G-I vs. G-III (temperature effect without melatonin); (**F**) D11 G-I vs. G-III.

KEGG enrichment analysis revealed distinct pathway profiles across temperature and melatonin treatments (Figure 7A–F). On day 4 at 35 °C (D4, G-I vs. G-II), significantly enriched pathways were limited to glycolysis/gluconeogenesis and motor proteins (Figure 7A). By day 11 (D11, Group-I vs. Group-II), a greater number of pathways became enriched, including neuroactive ligand–receptor interaction, phototransduction—fly, drug metabolism—cytochrome P450, metabolism of xenobiotics by cytochrome P450, propanoate metabolism, ascorbate and aldarate metabolism, porphyrin metabolism, retinol metabolism, and pentose and glucuronate interconversions (Figure 7B).

At 37 °C, early enrichment on D4 (Group-III vs. Group-IV) included Toll and Imd signaling pathway, arachidonic acid metabolism, longevity regulating pathway—multiple species, folate transport and metabolism, vitamin digestion and absorption, peroxisome, drug metabolism—cytochrome P450, and metabolism of xenobiotics by cytochrome P450 (Figure 7C). By D11, enriched pathways were dominated by ascorbate and aldarate metabolism, taurine and hypotaurine metabolism, uric metabolism—other enzymes, Toll and Imd signaling pathway, and metabolism of xenobiotics by cytochrome P450 (Figure 7D).

Temperature-only comparisons (Group-I vs. Group-III) also revealed heat-induced metabolic shifts. At day 4 (D4), significantly enriched pathways included neuroactive ligand–receptor interaction, phototransduction—fly, ether lipid metabolism, glycerophospholipid metabolism, alpha-Linolenic acid metabolism, thiamine metabolism, metabolism of xenobiotics by cytochrome P450, and drug metabolism—cytochrome P450 (Figure 7E). By day 11 (D11), pathway enrichment expanded to include alanine–aspartate and glutamate metabolism, valine–leucine and isoleucine biosynthesis, biosynthesis of unsaturated fatty acids, tyrosine metabolism, purine metabolism, tryptophan metabolism, D-Amino acid metabolism, motor proteins, phototransduction—fly, and neuroactive ligand–receptor interaction (Figure 7F). Taken together, these KEGG patterns indicate that melatonin treatment strongly engages detoxification and antioxidant-related pathways under heat stress. The activation of neuro—sensory, lipid, and P450-associated metabolic modules aligns with the observed improvements in sucrose responsiveness, enzyme activities, and survival. Together, these findings support the conclusion that melatonin facilitates metabolic stabilization and stress adaptation in honeybees exposed to elevated temperatures.

### 3.7. High Concordance Between RNA-seq and qRT-PCR Results Confirmed Consistent Gene Expression Patterns Under Heat Stress and Melatonin Treatment Across All Tested Genes

**Figure 8:**
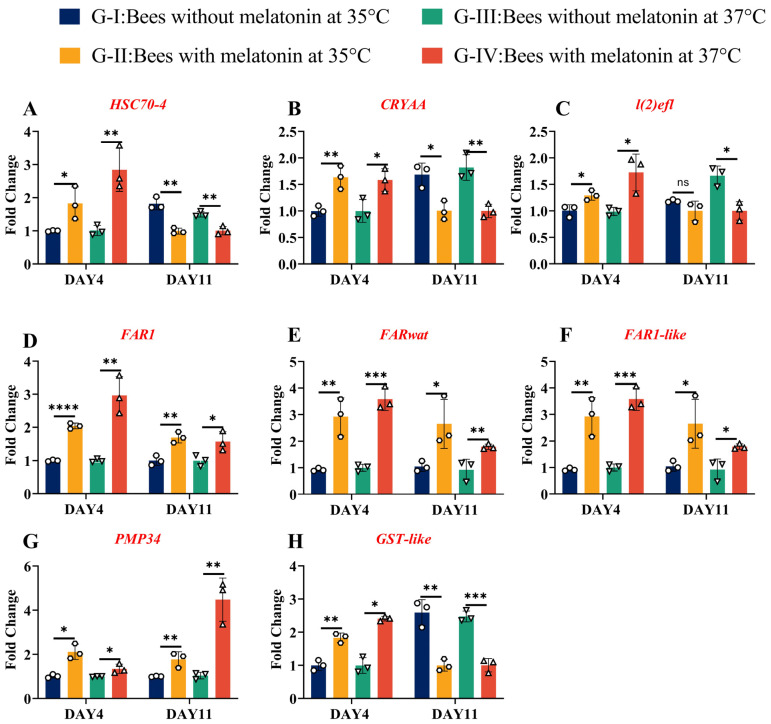
Validation of RNA-seq results by RT-qPCR. The fold change in transcript levels of genes involved in proteostasis and lipid/redox metabolism was quantified by RT-qPCR in adult *A. cerana* workers. Panels show (**A**) *FAR*-1, (**B**) *HSC70-4*, (**C**) *FAR-wat*, (**D**) *CRYAA*, (**E**) *PMP34*, (**F**) *l(2)efl*, (**G**) *FAR1-like*, and (**H**) *GST-like*. G-I, Bees without melatonin at 35 °C; G-II, Bees with melatonin at 35 °C (20 µg/mL); G-III, Bees without melatonin at 37 °C; G-IV, Bees with melatonin at 37 °C (20 µg/mL). Samples were collected on Day 4 and Day 11. The *y*-axis shows relative expression (fold change), calculated by the 2^−ΔΔCt^ method after normalization to the reference gene (β-actin) and expressed as n-fold difference relative to the 35 °C control group. The *x*-axis shows the four treatment combinations. Bars represent mean ± SD (*n* = 3 biological replicates). Asterisks denote statistically significant differences between melatonin-treated and control bees within each time–temperature combination (* *p* < 0.05, ** *p* < 0.01, *** *p* < 0.001, **** *p* < 0.0001; ns, not significant; two-tailed unpaired *t*-test).

To validate RNA-seq findings, a subset of differentially expressed genes involved in heat stress response, anti-dehydration, energy storage, and ROS scavenging was selected for RT-qPCR analysis (Table S4). *β-actin* and *GAPDH* were used as reference genes for normalization. High concordance was observed between RNA-seq and qRT-PCR results across all tested genes when *β-actin* was used as the reference gene (Figure 8). Similar expression patterns were also obtained when *GAPDH* was used as the reference gene (Figure S1). The corresponding statistical analysis data are provided in Table S5, further confirming the reliability of the transcriptomic data.

Heat shock proteins (*HSPs*) play a central role in insect thermal tolerance by functioning as ATP-independent or ATP-dependent molecular chaperones that prevent protein aggregation and facilitate refolding under stress [20]. Three HSP-related genes exhibited consistent melatonin-dependent regulation. Among them, Alpha-crystallin A (*CRYAA*), a small HSP (*sHSP*) originally identified in the eye lens, functions as an ATP-independent chaperone [38]. At day 4 (D4), *CRYAA* transcript levels were significantly elevated in melatonin-treated bees (Groups II and IV) compared to untreated controls (Group I and Group III) under both 35 °C (*p* = 0.0099) and 37 °C (*p* = 0.03). However, by D11, *CRYAA* expression declined markedly in all groups, with melatonin-treated bees showing lower levels than controls at both temperatures (35 °C: *p* = 0.01; 37 °C: *p* = 0.006) (Figure 8A). Lethal (2) -essential-for-life (*l(2)efl)*) [39], encoding an HSP20-family protein, was significantly upregulated at D4 in melatonin-fed bees at 37 °C (Group IV vs. Group III, *p* = 0.02). By D11, its expression dropped by approximately 50% relative to untreated bees (*p* = 0.01), suggesting a transient protective role during acute stress response (Figure 8B). Heat shock cognate 70-4 (*HSC70-4*), a classical ATP-dependent chaperone critical for protein homeostasis, was strongly induced by melatonin at Day 4 (D4). Transcript levels were approximately threefold higher in melatonin-treated bees than in controls at both 35 °C (*p* = 0.0447) and 37 °C (*p* = 0.009). Notably, under severe heat stress (37 °C), melatonin-treated bees (Group IV) showed about threefold higher *HSC70-4* expression than untreated bees (Group III; *p* = 0.005). However, by D11, *HSC70-4* expression in melatonin groups fell to roughly half of control bees at both temperatures (*p* < 0.01 for both), indicating a time-limited chaperone response (Figure 8C). Obviously, these results demonstrate that melatonin rapidly induces key HSPs during early heat stress but downregulates their pression at later stages, potentially reflecting effective mitigation of proteotoxic stress and a reduced need for sustained chaperone activity. Three fatty acyl-CoA reductase genes—*FAR1*, *FAR1-like*, and *FARwat*—involved in long-chain fatty alcohol synthesis and energy storage [40,41,42], showed consistent upregulation by melatonin across time points and temperature. *FAR1* transcript levels were significantly higher in melatonin-treated bees at D4 under both 35 °C (*p* < 0.0001) and 37 °C (*p* = 0.004), with expression approaching a threefold increase relative to control at 37 °C. This elevated expression persisted at D11 (35 °C: *p* = 0.005; 37 °C: *p* = 0.04) (Figure 8D). Similarly, *FAR1-like* and *FARwat*, both implicated in very long-chain fatty acid metabolism, exhibited parallel expression patterns, showing significant induction at D4 (35 °C: *p* = 0.002; 37 °C: *p* < 0.0001) and sustained upregulation at D11 (35 °C: *p* = 0.03; 37 °C: *p* = 0.004), albeit with modest declines over time and at higher temperature (Figure 8E,F). These findings suggest that melatonin promotes lipid biosynthesis and energy reserve accumulation, which may support prolonged survival and foraging activity under heat stress.

Two oxidative defense genes displayed distinct temporal dynamics in response to melatonin. Peroxisomal membrane protein 34 (*PMP34*), an adenine nucleotide transporter essential for peroxisomal protein import and redox homeostasis [43], was strongly upregulated by melatonin. At D4, *PMP34* expression was significantly higher in melatonin-treated bees at both 35 °C (*p* = 0.0058) and 37 °C (*p* = 0.05). Strikingly, the strongest induction occurred at D11 under severe heat stress, where melatonin-treated bees (Group IV) exhibited about 4-fold higher *PMP34* than Group III (*p* = 0.004), with significant upregulation also observed at 35 °C (*p* = 0.02) (Figure 8G). These patterns indicate a progressive enhancement of peroxisome-mediated antioxidant capacity. In contrast, glutathione S-transferase-like (*GST-like*), a key detoxification enzyme [2], showed early induction but late suppression. At D4, melatonin significantly increased *GST-like* expression at both 35 °C (*p* = 0.0024) and 37 °C (*p* = 0.04). However, by D11, *GST-like* transcript levels were reduced by nearly 50% relative to controls at both temperatures (35 °C: *p* = 0.003; 37 °C: *p* < 0.0001) (Figure 8H). This biphasic pattern aligns with the observed decline in total GST enzyme activity at D11 and suggests melatonin fine-tunes redox responses to avoid excessive or prolonged activation. Together, these molecular adaptations correlate with improved survival, gustatory responsiveness, and reduced oxidative damage, collectively explaining the colony-level benefits of melatonin supplementation under summer heat stress.

## 4. Discussion

Summer heat stress severely compromises honeybee colony performance by disrupting individual physiology, foraging behavior, and redox homeostasis [6,7,12,44,45]. In this study, we demonstrate that dietary melatonin, which is a phylogenetically conserved indoleamine with antioxidant and chronobiotic properties, confers multi-tiered protection against thermal stress in *A. cerana*. Melatonin supplementation not only enhanced colony weight gain during peak summer months but also preserved gustatory responsiveness (PER), reduced oxidative damage, and reprogrammed stress-responsive gene expression in a temperature and time-dependent manner. It should be noted that colony weight was the only colony-level parameter systematically recorded in the present field trial; however, future field studies should include additional measurements such as brood area, adult bee population, food stores, and queen performance to provide a more comprehensive evaluation of the colony-level benefits of melatonin supplementation. The improvement in colony biomass aligns with behavioral resilience. Melatonin-treated bees maintained higher sucrose sensitivity under both 35 °C and 37 °C, with the most pronounced effect at the higher temperature. Since PER strongly correlates with foraging motivation and nectar collection efficiency in honeybees [35], preservation of gustatory sensitivity may help sustain trophallaxis and resource influx during heat waves, processes that are critical for colony thermoregulation and brood rearing. Similar protective effects of melatonin on insect behavior have been reported in *Drosophila*, where melatonin attenuates age and stress-induced decline in locomotor activity [46,47]. At the cellular level, melatonin significantly suppressed ROS accumulation across all treatment groups, with the greatest relative reduction observed at 37 °C on day 11. This sustained antioxidant effect is consistent with melatonin’s well-documented role at both a direct free radical scavenger and an indirect regulator of antioxidant enzymes in insects [15,27,29,31]. Notably, melatonin enhanced GST activity under moderate heat (35 °C) while dampening excessive GST activation under severe heat stress (37 °C), reflecting a nuanced modulation that may prevent potential metabolic imbalance from excessive detoxification. This biphasic regulation mirrors findings in *Bombyx mori*, where melatonin fine-tunes GST and SOD activities to optimize redox balance under UV stress [48].Transcriptomic and qPCR validation further reveal that melatonin orchestrates a dynamic defense program. Early induction (D4) of small heat shock proteins (*CRYAA*, *l(2)efl*) and *HSC70-4* provides immediate proteostasis support—a strategy conserved across insects facing thermal stress [21]. The subsequent downregulation of these chaperones by D11 suggests successful resolution of proteotoxic stress, thereby avoiding the energetic cost of chronic *HSP* expression [13,20,49]. This “transient activation” model is supported by our observation that melatonin-treated bees exhibit lower ROS at D11 despite reduced HSP levels, indicating effective stress mitigation.

**Figure 9:**
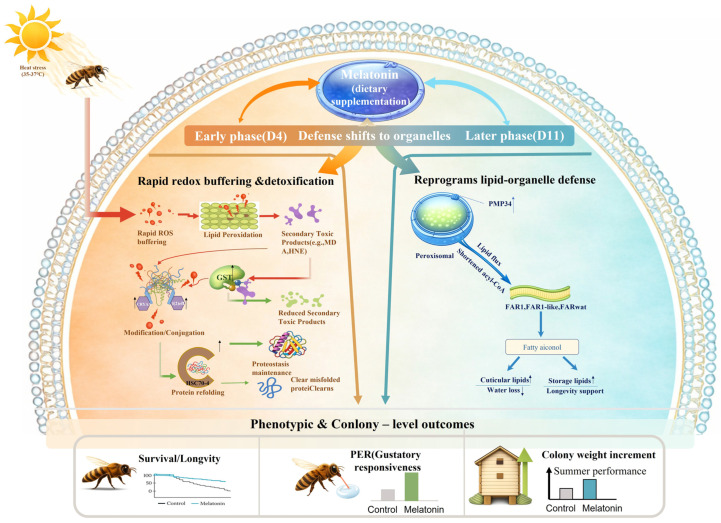
Biphasic mechanism by which melatonin enhances thermal resilience of *A. cerana*. In the schematic, red indicates enhanced stimulation, green lines indicate detoxification after treatment, and blue indicates sustained enhancement.

Concurrently, melatonin persistently upregulated fatty acyl-CoA reductases (*FAR1*, *FAR1-like*, *FARwat*), enzymes critical for synthesis of cuticular hydrocarbons and energy-storage lipids. Given that cuticular lipids are essential for desiccation resistance in bees [50], this metabolic shift likely enhances both longevity and dehydration tolerance during summer conditions, a dual benefit previously linked to lipid metabolism in heat-adapted *A. mellifera* populations. Most strikingly, melatonin differentially regulated ROS-scavenging systems over time. *GST-like* was transiently induced at early stages but suppressed at later time points, whereas *PMP34*, a peroxisomal transporter vital for redox compartmentalization, showed progressive upregulation, peaking at D11 under 37 °C. This pattern suggests a strategic shift from predominantly cytosolic to organelle-based antioxidant defense, optimizing long-term ROS management without disrupting redox signaling [36]. Together, these findings highlight melatonin not as a blunt antioxidant, but as a finely tuned homeostatic modulator of stress response networks. Most previous studies of heat stress in honey bees have focused on short-term exposures, such as transient treatments at extreme temperatures (25 °C or 45 °C for 1–5 h) [22,51]. In contrast, our study simulates a prolonged thermal challenge by maintaining bees at 37 °C over an extended period. Consistent with previous findings, we observed elevated reactive oxygen species (ROS) production and upregulation of antioxidant-related genes. However, our data also reveal distinctive features of heat stress response: ROS accumulation was not sustained continuously (Figure 5), and antioxidant gene regulation was non-monotonic, exhibiting dynamic, phase-dependent changes rather than a linear trend. These patterns suggest that bees actively modulate oxidative response to restore and maintain redox homeostasis while avoiding excessive or prolonged activation to maintain homeostasis [18]. Notably, the presence of melatonin appears to play a crucial role in facilitating this balanced redox state. Based on these observations, we propose a two-phase model describing how melatonin improves honey bee performance under heat stress (Figure 9). During the early phase of heat stress (Day 4), elevated ROS trigger lipid peroxidation, leading to the accumulation of secondary cytotoxic aldehydes such as malondialdehyde (MDA) and 4-hydroxynonenal (4-HNE) [50]. These reactive byproducts induce protein damage and misfolding [15,19,52]. Melatonin mitigates this oxidative and proteotoxic burden primarily by bolstering redox buffering capacity and detoxification pathways [28,29]—particularly through GST-mediated conjugation and clearance of lipid peroxidation-derived electrophiles [53], and synergistically supports proteostasis via molecular chaperones, such as *HSC70-4* [49], thereby rapidly restoring cellular homeostasis. As heat stress persists into the later phase (Day 11), the defensive strategy shifts toward organelle-level remodeling [54]. Melatonin promotes peroxisome–endoplasmic reticulum (ER) lipid flux and metabolic reprogramming [54], characterized by upregulation of peroxisomal membrane protein 34 (*PMP34*), enhanced β-oxidation [55], and fatty acyl-CoA reductase (*far*)-driven fatty alcohol biosynthesis [17,41]. This adaptive response fortifies cuticular lipid deposition to minimize water loss, while increasing energy-dense lipid storage to sustain long-term energy homeostasis and viability [21]. In summary, this biphasic mechanism, which comprises early-phase antioxidant/detoxification actions and late-phase lipid/organelle remodeling, provides a coherent explanation for the observed improvements in individual and colony-level performance, including extended lifespan, enhanced nectar collection ability, and increased colony weight under summer heat stress.

Concurrently, melatonin persistently upregulated fatty acyl-CoA reductases (*FAR1*, *FAR1-like*, *FARwat*), enzymes critical for synthesis of cuticular hydrocarbons and energy-storage lipids. Given that cuticular lipids are essential for desiccation resistance in bees [50], this metabolic shift likely enhances both longevity and dehydration tolerance during summer conditions, a dual benefit previously linked to lipid metabolism in heat-adapted *A. mellifera* populations. Most strikingly, melatonin differentially regulated ROS-scavenging systems over time. *GST-like* was transiently induced at early stages but suppressed at later time points, whereas *PMP34*, a peroxisomal transporter vital for redox compartmentalization, showed progressive upregulation, peaking at D11 under 37 °C. This pattern suggests a strategic shift from predominantly cytosolic to organelle-based antioxidant defense, optimizing long-term ROS management without disrupting redox signaling [36]. Together, these findings highlight melatonin not as a blunt antioxidant, but as a finely tuned homeostatic modulator of stress response networks. Most previous studies of heat stress in honey bees have focused on short-term exposures, such as transient treatments at extreme temperatures (25 °C or 45 °C for 1–5 h) [22,51]. In contrast, our study simulates a prolonged thermal challenge by maintaining bees at 37 °C over an extended period. Consistent with previous findings, we observed elevated reactive oxygen species (ROS) production and upregulation of antioxidant-related genes. However, our data also reveal distinctive features of heat stress response: ROS accumulation was not sustained continuously (Figure 5), and antioxidant gene regulation was non-monotonic, exhibiting dynamic, phase-dependent changes rather than a linear trend. These patterns suggest that bees actively modulate oxidative response to restore and maintain redox homeostasis while avoiding excessive or prolonged activation to maintain homeostasis [18]. Notably, the presence of melatonin appears to play a crucial role in facilitating this balanced redox state. Based on these observations, we propose a two-phase model describing how melatonin improves honey bee performance under heat stress (Figure 9). During the early phase of heat stress (Day 4), elevated ROS trigger lipid peroxidation, leading to the accumulation of secondary cytotoxic aldehydes such as malondialdehyde (MDA) and 4-hydroxynonenal (4-HNE) [50]. These reactive byproducts induce protein damage and misfolding [15,19,52]. Melatonin mitigates this oxidative and proteotoxic burden primarily by bolstering redox buffering capacity and detoxification pathways [28,29]—particularly through GST-mediated conjugation and clearance of lipid peroxidation-derived electrophiles [53], and synergistically supports proteostasis via molecular chaperones, such as *HSC70-4* [49], thereby rapidly restoring cellular homeostasis. As heat stress persists into the later phase (Day 11), the defensive strategy shifts toward organelle-level remodeling [54]. Melatonin promotes peroxisome–endoplasmic reticulum (ER) lipid flux and metabolic reprogramming [54], characterized by upregulation of peroxisomal membrane protein 34 (*PMP34*), enhanced β-oxidation [55], and fatty acyl-CoA reductase (*far*)-driven fatty alcohol biosynthesis [17,41]. This adaptive response fortifies cuticular lipid deposition to minimize water loss, while increasing energy-dense lipid storage to sustain long-term energy homeostasis and viability [21]. In summary, this biphasic mechanism, which comprises early-phase antioxidant/detoxification actions and late-phase lipid/organelle remodeling, provides a coherent explanation for the observed improvements in individual and colony-level performance, including extended lifespan, enhanced nectar collection ability, and increased colony weight under summer heat stress.

In conclusion, our findings identify melatonin as an integrative regulator that coordinates behavioral, physiological, and transcriptional adaptations to enhance honeybee thermotolerance. From an apicultural standpoint, melatonin supplementation delivered through sugar syrup or pollen patties offers a practical, low-cost strategy to bolster colony resilience under rising temperatures, complementing existing strategies like shade provision or hive ventilation. Although future field studies are needed to evaluate its performance under real-world conditions involving combined stressors (e.g., heat, pesticide exposure, etc.), the present results provide robust mechanistic evidence supporting the protective role of melatonin.

A two-phase model illustrating how melatonin improves honey bee performance under heat stress (Figure 9). During the early phase of chronic heat stress (Day 4), elevated ROS trigger lipid peroxidation, leading to the accumulation of secondary cytotoxic aldehydes such as malondialdehyde (MDA) and 4-hydroxynonenal (4-HNE). These reactive byproducts induce protein damage and misfolding. Melatonin mitigates this oxidative and proteotoxic burden primarily by bolstering redox buffering capacity and detoxification pathways—particularly through GST-mediated conjugation and clearance of lipid peroxidation-derived electrophiles, and synergistically supports proteostasis via molecular chaperones, notably *HSC70-4*, thereby rapidly restoring cellular homeostasis. As heat stress persists into the later phase (Day 11), the defensive strategy shifts toward organelle-level remodeling. Melatonin promotes peroxisome–endoplasmic reticulum (ER) lipid flux and metabolic reprogramming, characterized by upregulation of peroxisomal membrane protein 34 (*PMP34*), enhanced β-oxidation, and fatty acyl-CoA reductase (*far*)-driven fatty alcohol biosynthesis. This adaptive response fortifies cuticular lipid deposition to minimize water loss and simultaneously increases energy-dense lipid storage, thereby sustaining long-term energy homeostasis and viability under heat stress.

## Figures and Tables

**Table 1:** qPCR Primers used for validation.

Primers	Sequence (5′-3′)	Code Gene	Annealing Temperature (°C)	Reference
*FARwat*-F	GGTGGCATAAGAGAAAAATCGTTGT	**XM_017050809.3**	58	This paper
*FARwat*-R	ACGTTCCATTGGGCTATCGT			
*PMP34*-F	ATATCTGGTGCTGCTGGTGG	**XM_017051999.3**	58	This paper
*PMP34*-R	CGAGATCGCGTATTGTTGCC			
*FAR1-Like*-F	TTCAGGCCTTCCATGGTTGT	**XM_017062420.3**	58	This paper
*FAR*-Like-R	TGCTGTCGCTAACATTACTGGA			
*CRYAA*-F	TGGGGAGAGGATTTGAAGACAC	**XM_017050749.2** **XM** **_** **028667241.2**	58 58	This paper This paper
*CRYAA*-R *FAR1-F* *FAR1-R*	GCGAAAACAGCGATGAGGTC CGGTAGTGCGAGTTTCATTTGT CGCAGTGTTCGTTTACCCATT			
*l(2)efl*-F	GGGTGGCACATCGACCATTA	**XM_017053873.3**	58	This paper
*l(2)efl*-R	TGTTTCGCCTCTACCACGAC			
*Hsc70-4*-F	CCGCGCGTATCATACTCAGA	**XM_017053873.3**	58	This paper
*Hsc70-4*-R	GCCTTCGACATGTTTTATCGCTTT			
*GST-like*-F	CGTTGGCACGACCACAACAT	**XM_062073501.1**	58	This paper
*GST-like*-R	TTCAATGCACGAAGACCTGGA			
*β-actin*-F	TTCTTGCGGTGTCTCTTTGC	**XM_062077285.1**	58	This paper
*β-actin*-R	CTTTGCACATACCGGAGCCA			

## Data Availability

The RNA-seq raw reads generated in this study will be deposited in the NCBI Sequence Read Archive (SRA) upon acceptance of the manuscript; the accession number(s) will be provided at the proof stage. Processed data supporting the findings of this study, including differential expression results, are provided in the Supplementary Materials (Table S4).

## Supplementary Materials

The following supporting information can be downloaded at: https://www.mdpi.com/article/10.3390/insects17040379/s1, Table S1: Melatonin enhances colony weight increment during high temperature days; Table S2: Proboscis Extension Response (PER) Under Heat Stress and Melatonin Treatment; Table S3: Antioxidant and detoxification enzyme activities under heat stress and melatonin treatment, and the effects of melatonin on ROS scavenging and antioxidant defense in bees exposed to high temperatures; Table S4: Differential expression statistics for key genes in treatment (G2) versus control (G1) under heat stress at 35 °C and 37 °C on Day 4 and Day 11; Table S5: Melatonin upregulated the expression of *FAR1, FAR-wat, FAR1-like,* and *PMP34*, while bidirectionally regulating the heat shock protein- and glutathione S-transferase-related genes *HSC70-4, CRYAA, l(2)efl,* and *GST-like*; Figure S1: Validation of RNA-seq results by RT-qPCR. The fold changes in transcript levels of genes involved in proteostasis and lipid/redox metabolism were quantified by RT-qPCR in adult A. cerana workers. Panels show (A) FAR1, (B) HSC70-4, (C) FAR-wat, (D) CRYAA, (E) PMP34, (F) l(2)efl, (G) FAR1-like, and (H) GST-like. G-I, bees without melatonin at 35 °C; G-II, bees with melatonin at 35 °C (20 µg/mL); G-III, bees without melatonin at 37 °C; G-IV, bees with melatonin at 37 °C (20 µg/mL). Samples were collected on Day 4 and Day 11. The y-axis shows relative expression (fold change), calculated using the 2^−ΔΔCt^ method after normalization to the reference gene GAPDH and expressed as the n-fold difference relative to the 35 °C control group. The x-axis shows the four treatment combinations. Bars represent mean ± SD (n = 3 biological replicates). Asterisks denote statistically significant differences between melatonin-treated and control bees within each time–temperature combination (* *p* < 0.05, ** *p* < 0.01, *** *p* < 0.001, **** *p* < 0.0001; ns, not significant; two-tailed unpaired *t*-test).

## Abbreviations

The following abbreviations are used in this manuscript:

4-HNE	4-Hydroxy-2-nonenal (4-HNE)
AChE	Acetylcholinesterase
ANOVA	Analysis of variance
ATP	Adenosine triphosphate
AU	Arbitrary units
BCA	Bicinchoninic acid (BCA protein assay)
BLASTx	Basic Local Alignment Search Tool (translated nucleotide search)
BP	Biological Process
CarE	Carboxylesterase
CarEs	Carboxylesterases
CAT	Catalase
CC	Cellular Component
cDNA	Complementary DNA
CoA	Coenzyme A
CRYAA	Alpha-crystallin A chain (αA-crystallin)
CYP450	Cytochrome P450 monooxygenase
CYP450s	Cytochrome P450 monooxygenases
DAVID	Database for Annotation, Visualization and Integrated Discovery
DEGs	Differentially expressed genes
DNA	Deoxyribonucleic acid
ELISA	Enzyme-linked immunosorbent assay
ER	Endoplasmic reticulum
FAR	Fatty acyl-CoA reductase
FARwat	Fatty acyl-CoA reductase wat
FDR	False discovery rate
FPKM	Fragments per kilobase of transcript per million mapped reads
GAPDH	Glyceraldehyde-3-phosphate dehydrogenase
GeneRatio	Gene ratio (enrichment output metric)
GO	Gene Ontology
GraphPad	GraphPad Prism (software)
GST	Glutathione S-transferase
GST-like	Glutathione S-transferase-like
GSTs	Glutathione S-transferases
GTF	Gene Transfer Format (GTF annotation file)
HNE	4-Hydroxy-2-nonenal (HNE)
HSC70	Heat shock cognate 70
HSC70-4	Heat shock cognate 70-4
HSD	Honestly significant difference
HSP	Heat shock protein(s)
HSP20	Heat shock protein 20
HSPs	Heat shock proteins
KEGG	Kyoto Encyclopedia of Genes and Genomes
l(2)efl	Lethal (2) essential for life
MDA	Malondialdehyde
MF	Molecular Function
NCBI	National Center for Biotechnology Information
NO	Nitric oxide
nt	Nucleotide(s)
OH	Hydroxyl radical
ONOO	Peroxynitrite (ONOO−)
PBS	Phosphate-buffered saline
PANTHER	Protein Analysis Through Evolutionary Relationships
PCA	Principal component analysis
PCR	Polymerase chain reaction
PER	Proboscis extension response
Pfam	Protein families database (Pfam)
PMP34	Peroxisomal membrane protein 34 (PMP34)
qPCR	Quantitative polymerase chain reaction
qRT-PCR	Quantitative reverse transcription PCR
RefSeq	Reference Sequence (NCBI RefSeq database)
RH	Relative humidity
RNA	Ribonucleic acid
RNA-seq	RNA sequencing
RNS	Reactive nitrogen species
ROO	Peroxyl radical
RPKM	Reads per kilobase per million mapped reads
RSeQC	RNA-seq Quality Control (RSeQC package)
RT-PCR	Reverse transcription PCR
RT-qPCR	Quantitative reverse transcription PCR
SD	Standard deviation
SE	Standard error
SEM	Standard error of the mean
SOD	Superoxide dismutase
SPSS	Statistical Package for the Social Sciences
STAR	Spliced Transcripts Alignment to a Reference
StringTie	StringTie (transcript assembler)
SUPERFAMILY	SUPERFAMILY database
SWISS-PROT	UniProtKB/Swiss-Prot curated protein database
SYBR	SYBR Green (fluorescent dye used in qPCR)
TPM	Transcripts per million
TransDecoder	TransDecoder (coding region prediction tool)
TransGen	TransGen Biotech (reagent manufacturer)
UV	Ultraviolet
